# Enrofloxacin Pharmaceutical Formulations through the Polymer-Free Electrospinning of β-Cyclodextrin–oligolactide Derivatives

**DOI:** 10.3390/pharmaceutics16070903

**Published:** 2024-07-05

**Authors:** Diana-Andreea Blaj, Cătălina Anișoara Peptu, Maricel Danu, Valeria Harabagiu, Cristian Peptu, Alexandra Bujor, Lăcrămioara Ochiuz, Cristina Gabriela Tuchiluș

**Affiliations:** 1 “Petru Poni” Institute of Macromolecular Chemistry, 700487 Iasi, Romania; blaj.diana@icmpp.ro (D.-A.B.); hvaleria@icmpp.ro (V.H.); 2 Faculty of Chemical Engineering and Protection of the Environment, “Gheorghe Asachi” Technical University of Iasi, 700050 Iasi, Romania; catipeptu@yahoo.co.uk (C.A.P.); mdanu@tuiasi.ro (M.D.); 3 Faculty of Pharmacy, “Grigore. T. Popa” University of Medicine and Pharmacy, 700115 Iasi, Romania; alaxandra.bujor@umfiasi.ro; 4 Faculty of Medicine, “Grigore. T. Popa” University of Medicine and Pharmacy, 700115 Iasi, Romania; cristina.tuchilus@umfiasi.ro

**Keywords:** enrofloxacin, cyclodextrin–oligolactide, electrospinning, antibacterial activity, nanofibers

## Abstract

Enrofloxacin (ENR), a member of the fluoroquinolone class of antibiotics, is widely used in veterinary medicine to treat bacterial infections. Like many antibiotics, ENR has limited water solubility and low bioavailability. To address these challenges, drug formulations using solid dispersions, nanosuspensions, surfactants, cocrystal/salt formation, and inclusion complexes with cyclodextrins may be employed. The approach described herein proposes the development of ENR formulations by co-electrospinning ENR with custom-prepared cyclodextrin–oligolactide (CDLA) derivatives. This method benefits from the high solubility of these derivatives, enabling polymer-free electrospinning. The electrospinning parameters were optimized to incorporate significant amounts of ENR into the CDLA nanofibrous webs, reaching up to 15.6% by weight. The obtained formulations were characterized by FTIR and NMR spectroscopy methods and evaluated for their antibacterial activity against *Staphylococcus aureus*, *Escherichia coli*, and *Pseudomonas aeruginosa*. This study indicates that the presence of CDLA derivative does not inhibit the antibacterial activity of ENR, recommending these formulations for further development.

## 1. Introduction

The electrospinning method represents a versatile and effective approach for fiber preparation from diverse materials, including macromolecular or low-molecular-weight compounds [1,2]. Typically, the production of fibers requires high-molecular-mass polymers and concentrated solutions to ensure the necessary interchain interactions/entanglements and to prevent pulverization, which would otherwise yield particles instead of fibers. Cyclodextrins (CDs) and their derivatives are among the compounds suitable for electrospinning despite their low molecular weight, widely employed in pharmaceutical research [3]. It should also be mentioned that native and modified cyclodextrins are generally regarded as safe and are included in various pharmaceutical formulations across numerous countries worldwide [4]. Initially, research was focused on combining CDs with various polymers, such as polyesters [5,6,7], to produce nanofibers. CD–polyester fibers have proven effective for encapsulating various molecules, including vitamins [8], drugs [9,10,11], or enzymes [12,13,14], improving their stability during administration. Nevertheless, CDs can undergo direct electrospinning, forming nanofibers without the addition of polymers to the electrospinning solutions, a technique known as polymer-free CD electrospinning [15]. This approach proves especially advantageous in applications that require materials with increased amounts of CD in their composition for drug encapsulation and/or drug release modulation. Native CDs and their derivatives tend to self-organize partly due to intermolecular hydrogen bonds, forming layered aggregates, particles, two- and three-dimensional crystalline structures, and fibers in aqueous solutions, depending on their concentration [16]. The electrospinning of highly concentrated CD solutions, where increased hydrogen bond interactions determine significant aggregate generation, leads to the formation of nanofibers [17]. The effectiveness of CD electrospinning depends on the existence of aggregates and interactions among CD molecules in the feed solutions, resembling the entanglement interactions observed between polymer chains.

Due to the low water solubility of CD molecules, especially β-CD, their chemical modification is usually employed for solubility amelioration. The esterification of CDs leads to the formation of amphiphilic molecules with enhanced solubility in water, organic solvents, or their mixture favoring their interactions and intermolecular self-organization. Such interactions may give rise to nanoaggregates including micelles [18,19,20], spheres [18,21], vesicles [22,23], and spherical or rod-shaped particles [24]. Recent studies have shown that custom-synthesized α-, β-, and γ-cyclodextrin–oligolactide (CDLA) conjugates can also be utilized to produce nanofibers through electrospinning [25,26].

Cyclodextrin–oligoester (CDOE) derivatives represent an important and versatile class of biodegradable compounds, having properties that make them suitable for a wide range of applications (amphiphilic derivatives expected to enhance the delivery of drugs [18,19,20,21,22,23,24], building blocks for biodegradable polymer networks such as nanosponges or hydrogels [27,28,29], water purification [30], coatings for commercial polymers [31], initiators and catalysts for different polymerizations [32,33,34]). These derivatives are important due to their unique combination of properties, including biodegradability, biocompatibility, low toxicity, and the ability to form inclusion complexes with hydrophobic molecules [35]. This inclusion property is of particular importance in the pharmaceutical field, where it plays a crucial role in improving the photo- and thermostability and, in particular, the bioavailability of hydrophobic drugs. Therefore, various drugs (albendazole, acyclovir, lutein, cephradine, pindolol, amoxicillin, and lidocaine) have been encapsulated in esterified CDs with low substitution degrees [26,27,28,29,30,31,32,33,34,35,36,37,38,39,40].

The increased solubility of CDOE makes them particularly well suited for processing into nanofibers. An additional benefit of employing CD derivatives in the electrospinning process is their capacity to be co-electrospun with drug molecules, facilitating their formulation as fast-dissolving nanofibers [5,41,42,43]. In general, the association of CD with bioactive molecules gives rise to rapidly dissolving nanofibers, which are useful for various applications in the food, biomedical, and pharmaceutical fields [41]. Thus, the electrospinning process has been employed with various combinations of CDs and bioactive compounds to produce nanofibers. Well-established derivatives for such applications are considered hydroxypropyl-β-CD (HP-β-CD) [44,45,46,47,48,49,50,51,52,53,54,55,56] and hydroxypropyl-γ-CD [45,46,47,48,49,50,51,52,53,54,55], but methyl-β-CD [49,50,51,52,53,57] and sulfobutylether-β-CD [56,58,59] were also employed. The custom esterification of α-, β-, and γ-CD with oligolactide chains represents another approach for the preparation of curcumin-loaded electrospun nanofibers [26]. The structural characterization and antioxidant activity revealed that curcumin was best incorporated in β- and γ-CDLA nanofibrous webs, thus emphasizing the importance of CD/drug molecule complexation phenomena during the co-electrospinning process.

Various compounds with biological activity, including antibiotics, can also be encapsulated in CDs using the electrospinning process [3,43]. In general, the widespread use of antibacterial nanofibers is limited due to the low antibiotic loading capacity and the need to use toxic organic solvents to increase the loading capacity. Nanofibers based on bioderived excipients, such as CD, can incorporate a larger number of antibiotics while achieving better protection and physicochemical stability through encapsulation in the CD cavity. Thus, antibiotics such as chloramphenicol, ampicillin, kanamycin, and gentamicin have been incorporated into HP-β-CD nanofibers [60]. These antibacterial nanofibers dissolved rapidly in water and artificial saliva, releasing the CD/antibiotic complexes. The current study takes into consideration the amenability of enrofloxacin (ENR) for the preparation of electrospun pharmaceutical formulations. The development of the fluoroquinolone class of antibiotics was a breakthrough in the treatment of bacterial infections, having a broad spectrum of antibacterial activity against organisms that are resistant to many other substances. ENR is an antibiotic of this class that shows efficacy for veterinary use [61]. It can be used to treat specific infections against a broad spectrum of Gram-negative and Gram-positive bacteria in both the stationary and growth phases of bacterial replication [62]. However, the antibiotic’s poor water solubility and bitter taste hinder the development of effective pharmaceutical formulations [61,63]. Thus, to increase the water solubility and bioavailability of ENR, it was encapsulated in native CDs and HP-β-CD [64,65]. Among the CD complexes, ENR incorporation into β-CD had the highest stability constant [64]. However, the most significant improvement in ENR water solubility was obtained using HP-β-CD [64,65]. Another formulation pathway consists of the use of cyclodextrin covalent organic frameworks incorporated in electrospun thermoplastic polyurethane fibers, which showed promising potential in wound healing applications [66].

The approaches employed for the electrospinning formulation of antibiotics using CD derivatives took advantage of the outstanding solubility of HP-β-CD, which facilitates the preparation of highly concentrated solutions for successful polymer-free electrospinning and its ability to form inclusion complexes with drug molecules [3,5]. However, an emerging class of CDOE derivatives with controllable solubility properties in water and organic solvent mixtures has been found to meet polymer-free electrospinning requirements, notably the high concentration of the electrospinning solutions necessary to achieve the pseudo-entanglement conditions [25,26].

The current paper aims to expand the applicability of custom-prepared β-CDLA for ENR formulation as electrospun nanofibers. In the present study, ENR was incorporated into β-CDLA derivatives by electrospinning to obtain the corresponding fibers. The formation of fibers was followed by SEM microscopy, while the obtained nanofibers were subsequently evaluated by NMR and FTIR spectroscopy. The antimicrobial properties of the prepared nanofibers were also tested proving that ENR incorporation in nanofibers retains its toxicity against various microorganisms, including *Staphylococcus aureus*, *Escherichia coli*, and *Pseudomonas aeruginosa*.

## 2. Materials and Methods

### 2.1. Materials

β-Cyclodextrin (β-CD; Cyclolab, Hungary) was dried in an Abderhalden drying pistol with P_2_O_5_ under vacuum at 80 °C for 72 h and kept in a desiccator over P_2_O_5_, under an Ar atmosphere. D,L-lactide (LA; Purac, Amsterdam, The Netherlands) was recrystallized from toluene, dried under vacuum, and stored in a desiccator under an Ar atmosphere. The anhydrous dimethylformamide (DMF) and acetonitrile were purchased from Sigma-Aldrich, Saint Louis, MO, USA, while diethyl ether and methanol were purchased from VWR International (Vienna, Austria). The organocatalyst, 4-dimethylaminopyridine (DMAP), was acquired from Sigma-Aldrich, Saint Louis, MO, USA and used without purification. The α-cyano-4-hydroxycinnamic acid (CHCA) matrix, cationization agent (NaI), and Amberlyst 15 hydrogen form resin were purchased from Sigma-Aldrich, Saint Louis, MO, USA. Enrofloxacin was acquired from Selleckchem (Boston, USA) and used as received. The antimicrobial activity was assessed against standard microorganisms (American Type Culture Collection—ATCC): Gram-positive bacteria (*Staphylococcus aureus* ATCC 25923) and Gram-negative bacteria (*Escherichia coli* ATCC 25922 and *Pseudomonas aeruginosa* ATCC 27853).

### 2.2. Methods

Synthesis of β-cyclodextrin–oligolactide (β-CDLA): The β-CDLA derivatives were prepared using a slightly modified procedure that was previously published [26]. Briefly, the reaction was performed at room temperature (25 °C), in DMF, for 7 h, using a molar ratio of 1/8 β-CD/LA and 1/1 β-CD/DMAP. The β-CDLA products were purified by precipitation in cold diethyl ether after capturing the organocatalyst (DMAP) on Amberlyst 15 resin. Subsequently, the β-CDLA derivative was dried overnight under vacuum at 50 °C, resulting in a yield of 77%, and characterized by ^1^H NMR and MALDI MS.

β-CDLA: ^1^H NMR (400.13 MHz, DMSO-d6, δ, ppm): 5.85–5.69 (OH2, OH3), 5.48–5.47 (CH b′, OH b), 4.84 (H1, H1′), 4.51–4.18 (OH6, H6′, CH b), 3.92 (H5′), 3.85–3.80 (H3, 3′), 3.64 (H5, 6), 3.35 (H2, 2′, 4, 4′), 1.49–1.41 (CH3 a′), 1.30–1.29 (CH3 a). MALDI MS: 1930 g/mol.

Electrospinning: The electrospinning process was performed using a Spinbox electrospinning instrument (Bioincia—Valencia, Spain), controlled using the WinPumpTerm software, version 0.6. Highly concentrated solutions of β-CDLA/ENR (180–220% *w*/*v*) were tested, using a molar ratio of 1/1 between the components (considering the molecular mass determined by MALDI MS for β-CDLA). At the concentration of 220% *w*/*v*, simple β-CDLA fibers and β-CDLA/ENR fibers using a molar ratio of 2/1 were also prepared. All solutions were prepared in a 1/1 water/acetonitrile mixture (volume ratio). The electrospinning process was carried out at a flow rate of 0.25 mL/h, a needle-collector distance of 15 cm, and a voltage of 13 kV. The morphology of the electrospun materials was assessed using scanning electron microscopy. For comparison with fibers, physical mixtures of β-CDLA with ENR were prepared by short kneading, using molar ratios of 1/1 and 2/1.

### 2.3. Characterization

Solution viscosity: The dynamic viscosity of solutions was measured using a Physica MCR 501 rheometer (Anton Paar, Graz, Austria) with a 50 mm upper plate mounted in a parallel plate system. The flow curves were determined with shear rates ranging from 0.01 to 1000 s^−1^. Viscosity values are given for a shear rate of 100 s^−1^.

Fiber Morphology—Scanning Electron Microscopy: To investigate the formation of fibers, SEM images were recorded using a HITACHI SU 1510 scanning electron microscope (Hitachi SU-1510, Hitachi Company, Tokyo, Japan). All samples were fixed on an aluminum stub covered with a double adhesive carbon band. To obtain high-resolution images, the samples were placed on the chamber pedestal of a Cressington 108 Sputter Coater device (Cressington Scientific Instruments Ltd., Watford, UK) and coated with a 7 nm thick gold layer, under vacuum.

The average fiber diameter was estimated from SEM images using ImageJ 1.53k software, (LOCI, University of Wisconsin, Madison, WI, USA). Fiber diameter is expressed as average diameter ± standard deviation. To obtain an average diameter for each sample, over 70 fiber segments were randomly analyzed on 3 independently prepared samples.

Matrix-Assisted Laser Desorption/Ionization Mass Spectrometry: Mass spectra were registered using RapifleX MALDI TOF TOF MS (Bruker—Bremen, Germany). FlexControl 4.0 and FlexAnalysis 4.0 software (Bruker) were used to control the instrument and process the mass spectra. The β-CDLA sample was dissolved in 1 mL of water/acetonitrile mixture (1/1 *v*/*v*) and mixed using a Vortex-Genie 2 device (Scientific Industries, Inc, Bohemia, NY, USA). The CHCA matrix solution was prepared in a water/acetonitrile mixture (1/1 *v*/*v*) at a concentration of 20 mg/mL, while the NaI solution was prepared at a 5 mg/mL concentration in methanol. Samples were applied on the ground steel plate using the thin-layer method as follows: First, 1 μL of CHCA solution was applied to the MALDI MS target and left to dry at ambient conditions, followed by the deposition of 0.5 μL of sample solution (spiked with NaI) on top of the matrix layer, which was allowed to dry before analysis. The spectra were acquired in the positive reflectron mode. The laser ionization power was adjusted just above the threshold to produce consistent MS signals. The “partial sample” shooting mode, which covers a small area around the initial shooting site, was used to collect 18k spectra from different regions of the spot. The MS calibration was performed using poly(ethylene glycol) standards. The number average molecular mass was determined by MALDI MS using the following formula:

(1) $$Mn=\frac{\sum_{i}^{n}I_{i}\times m_{i}}{\sum_{i}^{n}I_{i}}$$

where

*I_i_*—monoisotopic peak intensity corresponding to the *m*/*z* ratio;
*m_i_*—*m*/*z* value of the corresponding *i* peak, with *z* = 1.

FTIR spectroscopy: Infrared spectra were recorded with a Bruker Vertex 70 FTIR spectrophotometer in an attenuated total reflection configuration. FTIR spectra were registered in the range of 600–4000 cm^−1^ at room temperature, employing a resolution of 4 cm^−1^ and accumulating 64 scans.

Nuclear Magnetic Resonance: The NMR spectra were recorded on a Bruker Avance NEO 400 MHz Spectrometer (Bruker, Rheinstetten, Germany) equipped with a 5 mm QNP direct detection probe and z-gradients. Spectra were recorded in DMSO-*d6* at room temperature using the standard parameter sets as provided by Bruker. The chemical shifts are reported as δ values (ppm) relative to the solvent residual peak (2.51 ppm for ^1^H).

The average length of oligolactide chains can be determined using the following formula:

(2) $$1+\frac{I_{{CH}_{3-a^{\prime }}}\;}{I_{{CH}_{3-a}}}$$

where

*I_CH3-a_*_′_ and *I_CH3-a_*—integral values for chain (1.49–1.44 ppm) and chain-end (1.31–1.29 ppm) methyl groups of attached oligolactide.

Antimicrobial assay: The antimicrobial activity was evaluated using the disk diffusion method [67,68]. Mueller–Hinton agar (Oxoid) and Mueller–Hinton agar Fungi (Biolab) were inoculated with the suspensions of the tested microorganisms: *Staphylococcus aureus* ATCC 25923, *Escherichia coli* ATCC *25922*, and *Pseudomonas aeruginosa* ATCC 27853. The turbidity of suspensions was adjusted to 0.5 McFarland standard, with a final turbidity of approximately 1 × 10^8^ CFU/mL. Sterile stainless-steel cylinders (5 mm internal diameter; 10 mm height) were applied on the agar surface in Petri plates. The tested compounds were β-CDLA/ENR 1/1 with 15.6% wt. ENR per dry sample, β-CDLA/ENR 2/1 with 7.8% wt. ENR per dry sample, and pure β-CDLA. In the first phase, all compounds were tested using solutions (prepared with water/DMSO in a ratio of 3/1 *v*/*v* as a solvent) having an equivalent concentration in enrofloxacin, i.e., 128 µg/mL, while the solution of pure β-CDLA was prepared in a concentration of 1.51 mg/mL. The wells were filled with 100 µL of the tested samples. The plates were left for 10 min at room temperature to ensure the equal diffusion of the sample in the medium and were then incubated at 35 °C for 24 h. Commercially available disks containing ciprofloxacin (CIP, 5 µg/disk) were also used for comparison. In each experiment, water/DMSO (3/1, *v*/*v*) was employed as a negative control. After 24 h of incubation, the diameters of inhibition were measured (in mm), and the results were noted. All tests were carried out in triplicate. The results are expressed as mean ± standard deviation.

## 3. Results and Discussion

**Scheme 1:**
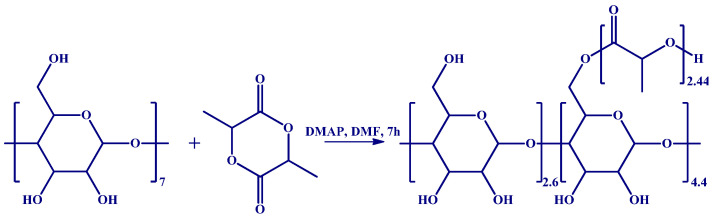
β-CDLA synthesis.

The custom-prepared CD derivatives employed in this study should have high solubility in water/organic solvents and retain the ability to form inclusion complexes. Previously, we found that β-CDLA derivatives may be successfully employed for the co-electrospinning of curcumin provided that the substitution degree with oligolactide chains is maintained at a low level. Therefore, β-CDLA was prepared through ring-opening oligomerization of D,L-lactide initiated by β-CD and catalyzed by a combined system, comprising DMAP organocatalyst and monomer inclusion activation (Scheme 1) [26,69,70,71].

**Figure 1:**
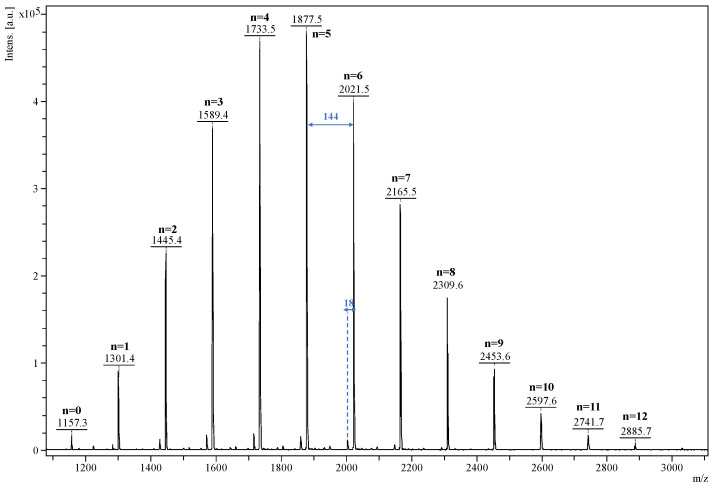
MALDI MS spectrum of β-CDLA showing the oligolactide characteristic 144 Da sequence of the main series and a minor series downshifted with 18 Da.

The β-CDLA product was analyzed by MALDI MS to qualitatively describe the mass spectrum of the resulting derivative and to quantify the molecular mass. The mass spectrum depicted in Figure 1 shows a main series of peaks described by the following equation: *m*/z = 1134 (β-CD) + n*144 (dilactate) + 23 (Na^+^). This series is associated with the direct ring-opening of the lactide in the presence of β-CD, the compound having the structure represented in Scheme 1. However, common side reactions in the ring-opening of lactide monomers are transesterification exchanges (intermolecular and backbiting) that may lead to the formation of another peak series shifted with 72 Da, corresponding to the mono-lactate unit [69,72]. This series has an odd number of mono-lactate units, which may be identified in the MALDI mass spectrum at a very low intensity, close to the background noise. This signifies that transesterification exchanges were kept to a minimum using these particular reaction conditions. Besides the main series, a lower-intensity one, downshifted with 18 Da, can be also observed, which is determined using the following equation: *m*/z = 1134 (β-CD) + n*144 (dilactate) + 54 (acrylate) + 23 (Na^+^). This series is a consequence of water elimination from the oligolactide end chains, thus resulting in the formation of the acrylate-terminated β-CDLA derivatives. The water elimination reaction was previously detected by MALDI MS and confirmed by MS/MS fragmentation studies [69]. Following the qualitative characterization, the mass spectrum of the β-CDLA product was employed to determine the molecular mass. Using Equation (1), the calculated average numerical molecular mass was found to be 1930 g/mol, indicating the attachment of a total number of 5.37 lactide (10.74 mono-lactate) units to β-CD. Although MALDI MS analysis might be biased when representing the molecular mass values, the potential error may be neglected for the low-dispersity β-CDLA derivatives, as was previously demonstrated by the excellent agreement between MALDI MS kinetics and ^1^H NMR measurements of lactide conversion [69]. Unfortunately, the amount of lactide attached to the CD was difficult to calculate using ^1^H NMR spectroscopy because of peak overlapping.

**Figure 2:**
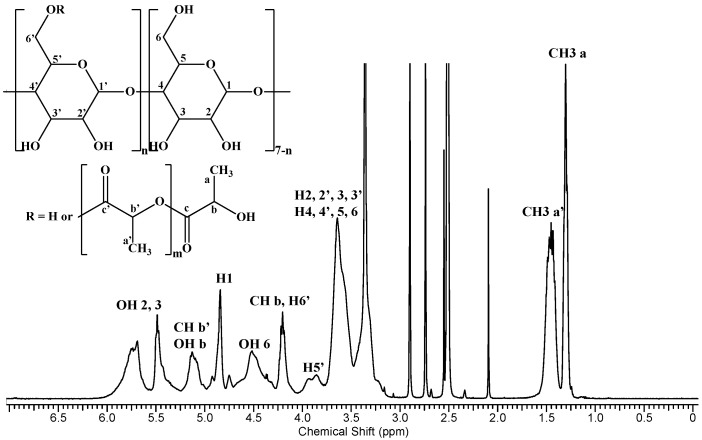
^1^H NMR spectrum of β-CDLA.

The ^1^H NMR analysis of the β-CDLA derivative revealed the average length of the attached oligolactide chain and the substitution site position on the β-CD molecule. Thus, utilizing data derived from the ^1^H NMR spectrum (Figure 2) and Equation (2) described in the experimental section, which involves the integrals for the protons corresponding to in-chain and end-chain methyl groups (a and a’), an average chain length of 2.44 mono-lactate units was determined. Moreover, by dividing the total substitution degree resulting from MALDI MS by the average chain length obtained from NMR spectroscopy, an average number of 4.4 substitution sites was obtained. Previous NMR studies on β-CDLA synthesis in bulk [73] or solution conditions [26,69], through ^13^C DEPT 135 NMR, indicated that the substitution occurs at the primary hydroxyl groups of the β-CD molecule, probably because of intramolecular exchanges on the CD molecule [71]. Therefore, we may consider the structure of the synthesized β-CDLA to correspond to the one depicted in Scheme 1.

### 3.1. Electrospinning

Nanofibers based on β-CDLA and ENR were prepared considering the previous results obtained both on the preparation of β-CDLA nanofibers using a water/acetonitrile mixture [25] and on the incorporation of curcumin into CDLA fibers, using water/ethanol [26]. The β-CDLA solution concentration necessary for fiber separation was found to be significantly influenced by the presence of the drug in the feed. Previously, in the case of curcumin co-electrospinning we noticed an increase in the β-CDLA solution concentration, necessary to accommodate the additional drug molecules in the feed mixture.

**Scheme 2:**
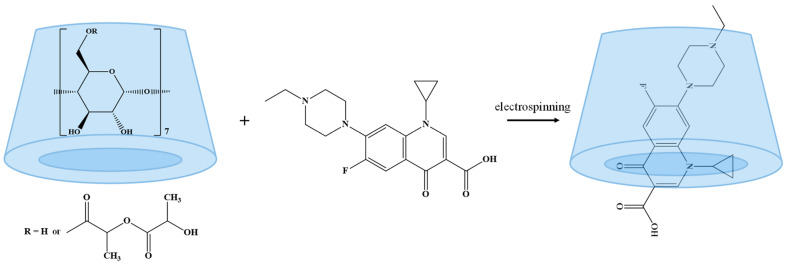
Preparation of the β-CDLA/ENR formulations through polymer-free electrospinning.

The amphiphilic nature of β-CDLA, due to the carbohydrate and oligolactide parts of the molecule, restricts the range of solvents that may be used at the concentration values required for a successful electrospinning process that leads to fiber formation. The conducted experiments (Table 1) involved the utilization of a water/acetonitrile (1/1 *v*/*v*) mixture for the electrospinning process of highly concentrated β-CDLA/ENR solutions (Scheme 2). This solvent combination proved to be excellent for the electrospinning of β-CDLA, leading to thinner fibers as compared to the DMF solvent [25], as well as providing an excellent environment for ENR solubilization [74].

Previous studies related to the electrospinning of β-CDLA derivatives with curcumin [26] revealed that a minimal concentration of 180% *w*/*v* was necessary for nanofiber formation. On the other hand, the low solubility of ENR represents another challenge for the electrospinning process, as the introduction of a significant drug amount is needed due to the high concentration of β-CDL in the feed solution. Studies in the literature indicate that the solubility of ENR in water is approximately 0.23 g/L, while in acetonitrile, it is approximately 7.7 g/L. Taking into consideration the ENR drug formulation through an electrospinning procedure, the main interest is related to the solubility increase in the feed solution conditions, water/acetonitrile 1/1 *v*/*v*. Theoretically, the solubility of ENR alone in water/acetonitrile 1/1 *v*/*v* should not exceed 3.9 g/L. However, when β-CDLA was introduced in a 1/1 molar ratio to ENR, the solubility increased about 85.8 times for the 180% *w*/*v* solution, 95.3 times for the 200% *w*/*v*, and 105 times for 220% *w*/*v*. Such results are in agreement with the studies that revealed an ENR solubility increase in water solutions of HP-β-CD [75].

**Figure 3:**
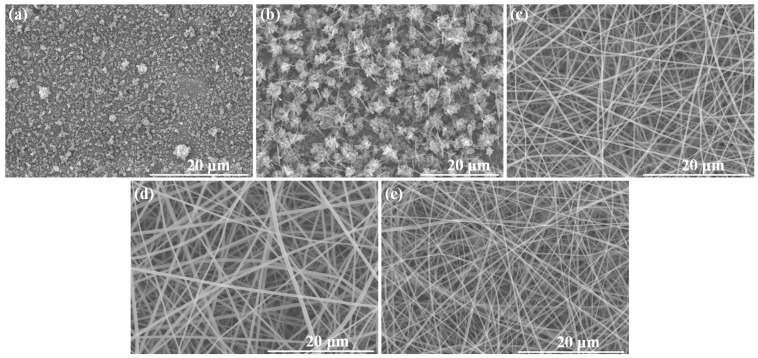
SEM images for β-CDLA/ENR at concentrations: (**a**) 180% *w*/*v*, (**b**) 200% *w*/*v*, (**c**) 220% *w*/*v*, using a 1/1 molar ratio, (**d**) 220% *w*/*v* using a 2/1 molar ratio, and (**e**) β-CDLA 220%.

Therefore, for the first attempt to obtain ENR/CDLA nanofiber formulations, a 180% *w*/*v* β-CDLA solution concentration was employed, to which an ENR amount corresponding to a 1/1 β-CDLA/ENR molar ratio was added. However, the effect of the ENR presence in the electrospun mixture resulted in particle formation (Figure 3a). Consequently, a more concentrated solution in β-CDLA is required to prepare nanofibers. The increase in the feed solution to 200% *w*/*v* (with a corresponding amount of ENR) resulted in a mixture of short fibers and particles (Figure 3b), indicating a certain trend toward fiber formation. Consequently, a further increase to a 220% *w*/*v* concentration led to the successful formation of relatively uniform fibers, with an average diameter of 342 ± 59 nm (Figure 3c).

Fibers were also prepared using a molar ratio of 2/1 β-CDLA/ENR at a concentration of 220% *w*/*v* (Figure 3d), exhibiting diameters of 377 ± 92 nm, which is relatively similar to that obtained with the 1/1 molar ratio. Notably, reducing the ENR amount slightly influences the electrospinning process by increasing the fibers’ average diameter and also the standard deviation. β-CDLA fibers without ENR having average fiber diameters of 307 ± 77 nm (Figure 3e), obtained at 220% *w*/*v* solution concentration, were also prepared for further structural characterization comparison.

The increase in solution concentration necessary for the formation of nanofibers is attributed to the participation of ENR in the polymer-free electrospinning process, which may disrupt the hydrogen bonding entanglement during the electrospinning process [76,77]. The electrospinnabillity of the CD derivatives was also assessed based on the viscosity of the feed solutions. Thus, the concentration increase corresponds to a specific variation in the viscosity values in accordance with the pseudo-entanglement conditions. In the case of CDs without polymers, the hydrogen bond interactions led to a higher viscosity increase rate once the electrospinnabillity concentration value was reached. We noticed that a certain increase in the measured viscosity values was associated with the increase in concentration, from 1.85 (180% *w*/*v*) to 3.04 (220% *w*/*v*) values. However, the solution of pure β-CDLA at 220% *w*/*v* concentration had a viscosity value of 1.84, almost similar to the solution containing CDLA/ENR 1/1 at 180% *w*/*v*. Also, the solution containing CDLA/ENR 2/1 at 220% *w*/*v* had a 2.62 viscosity value. Thus, we may conclude that the presence of ENR increases the overall solution viscosity and, at the same time, diminishes its electrospinnabillity. Nevertheless, it is worth noting that ENR formulation through co-electrospinning with β-CDLA allowed for the preparation of nanofibrous webs with the drug molecules homogeneously dispersed at a molecular level. The drug content in such pharmaceutical formulations could reach 15.6% wt. (the weight content for each formulation is given in Table 1). For comparison, the ENR amount loaded in cyclodextrin covalent organic frameworks reached approximately 4.75% wt. [66].

### 3.2. Characterization

**Figure 4:**
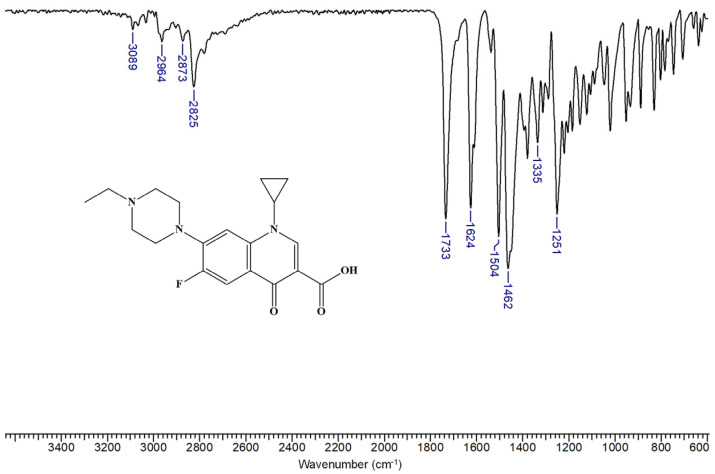
FTIR spectrum of enrofloxacin.

β-CDLA and β-CDLA/ENR fibers and the corresponding physical mixtures were characterized and compared by FTIR spectroscopy. Generally, FTIR proves valuable in confirming the formation of CD–drug inclusion complexes, as evidenced by spectral changes such as the decrease, shift, or disappearance of specific IR absorption bands. The FTIR spectrum of ENR is similar to those in the literature [65] (Figure 4). ENR has absorption bands in the region 3089–2825 cm^−1^ due to O-H (from the carboxyl group) and C-H stretching vibrations. Also, at 1733 cm^−1^, a strong absorption band is observed, corresponding to the C=O stretching vibration from the carboxyl group substituted in the α position. The band at 1624 cm^−1^ can be attributed to the C=O stretching vibration of the quinolinone with partial enol character. The bands at 1504 and 1462 cm^−1^ are associated with the C=C stretching vibration of the aromatic moiety. Absorption bands are also observed at 1335 and 1251 cm^−1^ due to the stretching vibrations of the C (aromatic)-F bond and the C-N bond, respectively, in tertiary amines.

**Figure 5:**
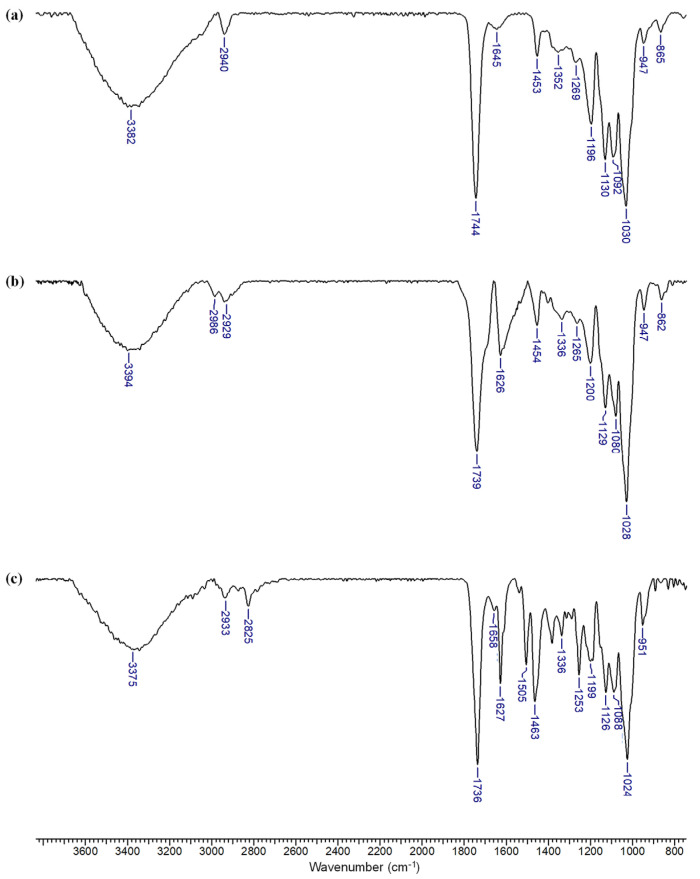
FTIR spectra of (**a**) β-CDLA fibers, (**b**) β-CDLA/ENR fibers, and (**c**) β-CDLA/ENR physical mixture, using a 2/1 molar ratio.

The FTIR spectrum of the β-CDLA nanofiber (Figure 5a) highlights specific bands attributable to both the β-CD and oligolactide components, which align with previously reported data [26]. More precisely, the band at 3382 cm^−1^ corresponds to O-H stretching vibrations, arising from unreacted hydroxyl groups in the β-CD molecule, the oligolactide chain, or associated water molecules. A weaker C-H stretching vibration is observed at 2940 cm^−1^, which is associated with both β-CD and oligolactide chains, while the strong absorption band at 1744 cm^−1^ indicates the C=O stretching vibrations due to oligolactide ester bonds. At 1645 cm^−1^, the H-O-H deformation band of water is noticeable. The oligolactide chain exhibits several characteristic bands: a C-H bending vibration at 1453 cm^−1^, an O-H bending vibration at 1352 cm^−1^, and a C-O-C stretching vibration at 1196 cm^−1^. Additional C-O stretching vibrations are detected at 1269, 1092, and 947 cm^−1^. The carbohydrate component of β-CDLA is associated with the presence of strong absorption bands at 1130 cm^−1^ (C-O stretching vibration), 1030 cm^−1^ (C-O-C stretching vibrations), and a weaker band at 865 cm^−1^ (C-O-C stretching vibrations).

Regarding ENR incorporation into fiber formulations or physical mixtures, using a 2/1 molar ratio, differences are mostly noted in the 1750–1250 cm^−1^ region (Figure 5b,c). The bands specific to ENR are discernible only at 1626 and 1336 cm^−1^, particularly in the case of fibers. However, other bands in this spectral region overlap with those of β-CDLA, such as bands found at wave numbers 1453 and 1265 cm^−1^, respectively (Figure 5b). The characteristic bands of β-CDLA are more pronounced given their higher amount compared to ENR, thus hindering the direct observation of the bands associated with the active compound in the nanofibers. However, ENR encapsulation in β-CDLA nanofibrous formulations may be ascertained by taking into consideration the specific change in the shape of the bands at 1626 and 1336 cm^−1^, which is modified due to the interactions with β-CDLA. In contrast, the corresponding physical mixture shows absorption bands at 1627, 1505, 1463, 1336, and 1253 cm^−1^ (Figure 5c), similar to ENR, without the specific shape changes remarked in the fiber’s case. The band corresponding to the C=O vibration undergoes a shift to lower wavenumbers in both ENR-loaded fibers (1739 cm^−1^) and the physical mixture (1736 cm^−1^). Therefore, it is evident that in the physical mixture, both components retain their characteristic bands, whereas in the case of the fibers, the bands exhibit changes due to interactions between the two components. These observations suggest the associations between β-CDLA and ENR that emerge after electrospinning, probably involving the formation of an inclusion complex. Similar phenomena were generally observed for CD derivative–drug electrospun formulations [26,55,58,60].

**Figure 6:**
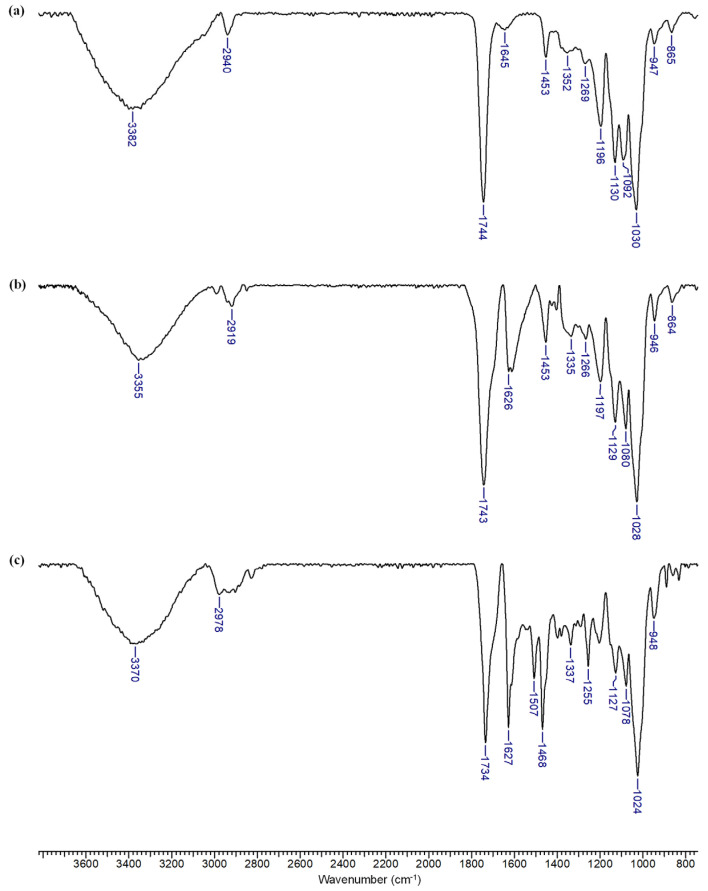
FTIR spectra of (**a**) β-CDLA fibers, (**b**) β-CDLA/ENR fibers, and (**c**) β-CDLA/ENR physical mixture, using a 1/1 molar ratio.

More pronounced spectral changes are observed when the ENR amount is increased in the electrospinning feed solution. The nanofiber formulations present clear bands associated with the bioactive compound at 1626 and 1335 cm^−1^ for the 1/1 β-CDLA/ENR molar ratio (Figure 6b). At 1453 and 1266 cm^−1^, the absorption bands of ENR and β-CDLA overlap. On the other hand, in the FTIR spectrum of the β-CDLA/ENR physical mixture, bands associated with ENR are observed at multiple wavenumbers, specifically at 1627, 1507, 1468, 1337, and 1255 cm^−1^ (Figure 6c). Also, the band at 1734 cm^−1^ corresponding to the C=O stretching vibration, a group common to both compounds, is shifted in the physical mixture to a lower wavenumber compared to the ENR-loaded β-CDLA fibers. The bands corresponding to β-CDLA are less predominant in the spectrum of the physical mixture probably because of the heterogeneous distribution.

**Figure 7:**
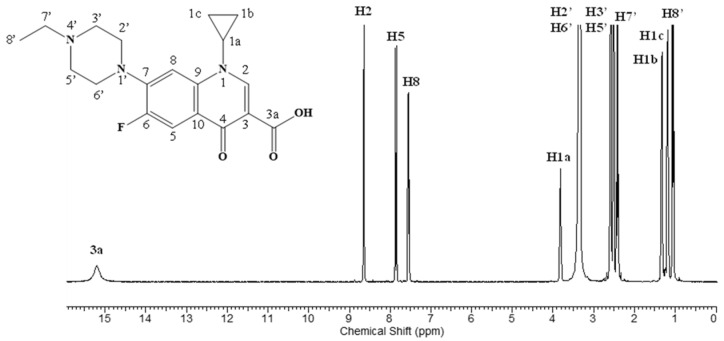
^1^H NMR spectrum of enrofloxacin.

NMR spectroscopy was also employed for the characterization of ENR-loaded nanofibers. Initially, the ^1^H NMR spectrum of ENR was recorded for comparison with the β-CDLA/ENR nanofibers (Figure 7).

**Figure 8:**
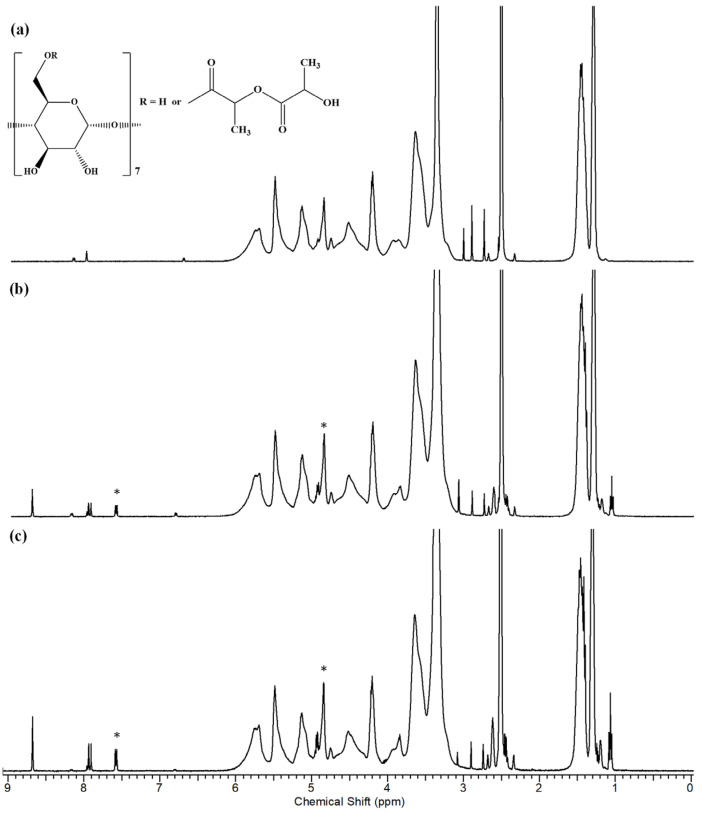
^1^H NMR spectra for (**a**) β-CDLA fibers, (**b**) β-CDLA/ENR fibers (2/1 molar ratio), and (**c**) β-CDLA/ENR fibers (1/1 molar ratio); the symbol * indicates the peaks taken into account for the calculation of the CDLA/ENR molar ratio.

The ^1^H NMR analysis reveals that the structure of the β-CDLA derivatives remains intact after the electrospinning process (Figure 8a). The ^1^H NMR spectra indicate the presence of ENR in nanofibers obtained through electrospinning, for both molar ratios (Figure 8b,c). Furthermore, NMR analysis was employed to ascertain the molar ratio of β-CDLA to ENR in the nanofibers. By assessing the ratio of the integrals of the anomeric protons in β-CDLA and the H8 proton of ENR, it was determined that the molar ratio was maintained after the electrospinning process in both cases. Additionally, Table 2 shows the ^1^H NMR peak values for pure ENR and ENR formulations with β-CDLA at both molar ratios. The comparative analysis reveals small downfield chemical shifts (under 0.1 ppm), suggesting interactions between ENR and β-CDLA.

### 3.3. Antibacterial Properties

Various types of cyclodextrin derivatives were employed in combination with ENR and showed increased drug bioavailability due to the formation of inclusion complexes with improved water solubility [64,65,75]. However, no comparison was made with the activity of the pure drug possibly because of the consistent difference in solubility (e.g., HP-β-CD improved ENR water solubility 916-fold [65]). On the other hand, ENR-loaded β-cyclodextrin covalent organic frameworks were mounted on stable electrospun mats and revealed satisfactory antibacterial performance attributed to the sustained release of enrofloxacin [66]. These properties recommend this ENR delivery device for wound healing applications.

**Figure 9:**
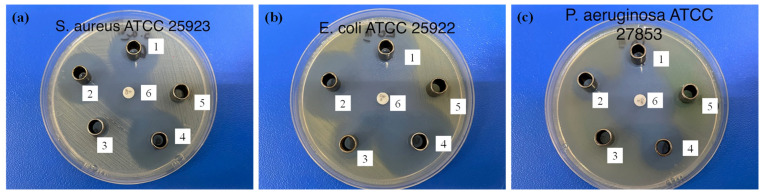
Antibacterial activity of the tested samples against (**a**) *S. aureus*, (**b**) *E. coli*, and (**c**) *P. aeruginosa*: 1—β-CDLA/ENR 1/1, 2—β-CDLA/ENR 2/1, 3—β-CDLA nanofibers, 4—ENR, 5—water/DMSO (3/1 *v*/*v*), 6—ciprofloxacin.

The antibacterial activity of the β-CDLA-based nanofiber ENR formulations was performed through the disk diffusion assay utilizing both Gram-positive (*Staphylococcus aureus*) and Gram-negative (*Escherichia coli* and *Pseudomonas aeruginosa*) bacteria. ENR content in each sample was kept similar. The diameter of the inhibition zone for each tested sample (expressed in mm) is listed in Table 3. The antibacterial activity against the Gram-positive *S. aureus* bacteria following a 24 h exposure period is illustrated in Figure 9. The β-CDLA/ENR nanofibers with different molar ratios between the components showed obvious inhibition zones due to the suppression of bacteria growth in this region. The external diameters of the inhibition zones were 26.10 ± 0.05 mm and 27.00 ± 0.00 mm, respectively, for β-CDLA/ENR 1/1 and 2/1. The β-CDLA nanofiber without ENR used as a reference did not exhibit an inhibition zone and consequently showed no antibacterial activity. A water/DMSO (3/1 *v*/*v*) mixture, which was employed for complete sample dissolution, was also used as a control, and as expected, no inhibition zone was observed. For comparison, pure ENR was also tested against *S. aureus*, yielding a diameter of the inhibition zone of 29.00 ± 0.00 mm. The β-CDLA/ENR-based nanofibers were also compared with the activity of the ciprofloxacin standard disk. Considering the inhibition zone diameter of this antibiotic against Gram-positive bacteria, pure ENR showed slightly higher activity, while β-CDLA/ENR samples exhibited comparable antibacterial activity.

The observed inhibition values for ENR-containing samples were relatively similar, slightly lower for the samples containing β-CDLA. Most probably this small difference is due to inclusion phenomena that may cause a delay in the antibacterial activity of ENR. In fact, in aqueous solutions, CD complexation has been shown to increase the stability of dissolved drugs, but in some situations, CD may lead to undesired degradation [78]. However, such studies were not considered for water/organic solvent mixtures. The testing conditions employed herein required the full solubility of the pure ENR sample for comparison reasons. Possibly, in the water/ENR dispersion, where ENR solubility is much lower, the effect of β-CDLA would significantly enhance the antibacterial activity of ENR.

The antibacterial activity of β-CDLA/ENR nanofibers against Gram-negative bacteria was also tested against *E. coli* and *P. aeruginosa*. The β-CDLA/ENR 2/1 sample yielded the highest inhibition zone against *E. coli*, having a diameter of 35.10 ± 0.05 mm. This antibacterial activity was slightly higher than the one determined for pure ENR (34.10 ± 0.05 mm) and better than the inhibition observed using β-CDLA/ENR 1/1 (31.70 ± 0.06 mm) and ciprofloxacin standard (31.00 ± 0.00 mm). As expected, the water/DMSO mixture did not exhibit any antibacterial activity. In contrast, pristine β-CDLA nanofibers presented significant activity against *E. coli* (15.00 ± 0.00 mm).

Regarding the effect of the β-CDLA presence, the effect of pristine β-CDLA against *E. coli* was surprising. However, previous studies revealed the toxicity effect of CDs and their derivatives against Gram-negative bacteria [79]. This activity was found to occur for CD derivatives due to a combination of encapsulation capacity and surface interactions, which may provide the capacity to effectively disrupt the cell membrane and wall. This effect did not seem to be cumulative with the antibacterial effect of ENR, since the observed inhibition values were rather similar.

The antibacterial activity of the β-CDLA/ENR nanofibers was significantly lower against the other Gram-negative bacterium (*P. aeruginosa*). The β-CDLA/ENR nanofibers with a 2/1 molar ratio (18.00 ± 0.00 mm) exhibited a slightly higher inhibition than those with a 1/1 molar ratio (17.10 ± 0.05 mm), both being significantly lower than the ciprofloxacin standard (32.30 ± 0.57 mm). Out of the samples containing ENR, the highest activity was registered for the pure ENR antibiotic (21.00 ± 0.00 mm). The water/DMSO solvent mixture and β-CDLA references showed no antibacterial activity.

Overall, we may note that the β-CDLA/ENR formulations containing a relatively high amount of active principle (15.6% wt. for 1/1 molar ratio and 7.8% wt. for 2/1 molar ratio between components) did not affect significantly the activity against Gram-positive and Gram-negative bacteria. Moreover, the inhibition zones closely matched the pure ENR and ciprofloxacin standard, except *P. aeruginosa*, where ciprofloxacin exhibited superior antibacterial efficacy. Our results show the possibility of obtaining a dry formulation of enrofloxacin with fast dispersion capacity in aqueous solutions due to the nanofibrous nature and lack of covalent crosslinking, similar to the work of Topuz et al. [60]. CD-based antibiotic formulations, such as HP-β-CD nanofibers, which are loaded with substantial amounts of antibiotics (over 10% wt. of ampicillin, kanamycin, and gentamicin [60]) showed remarkable activity against *E. coli*. However, the fast dispersion capacity does not necessarily ensure the increase in the drug effect due to interference of the complexation, and a comparison with the pristine drug would be necessary. Nevertheless, the antibacterial activity of the tested formulations can be modulated using high concentrations of the antibiotic.

## 4. Conclusions

The custom-prepared β-cyclodextrin–oligolactide (CDLA) derivative employed in this study was structurally assessed using MALDI MS and NMR, revealing an average of 4.4 chains with an average of 2.44 lactate units, at the smaller rim. The optimal β-CDLA concentration necessary to successfully incorporate ENR was 220% *w*/*v*, indicating that the presence of the drug in the electrospinning feed solution affects the hydrogen bonding entanglements. However, the average fiber diameters remained between 300 and 400 nm and were not significantly affected by the presence of ENR. FTIR analysis revealed significant band shifts in the ENR electrospinning formulations, suggesting physical interactions (likely inclusion) with β-CDLA. NMR analysis of the ENR-loaded nanofibrous webs confirmed that the drug amount in the feed solutions was maintained after the electrospinning process. ENR formulations were effective against both Gram-positive and Gram-negative bacteria, showing similar levels of antibacterial activity compared to pure ENR and ciprofloxacin. Additionally, tests on pure β-CDLA revealed moderate activity against *E. coli*, likely due to membrane disruption through physical inclusion phenomena.

## Figures and Tables

**Table 1:** Parameters used for electrospinning β-CDLA/ENR systems and average fiber diameters.

Composition	β-CDLA/ENR Molar Ratio	CDLA Concentration (*w*/*v*%)	ENR Amount (% wt.)	Viscosity (±0.05 Pa·s)	Morphology	Diameter (nm)
β-CDLA/ENR	1/1	180	15.6	1.85	particles	-
β-CDLA/ENR	1/1	200	15.6	2.34	fibers, particles	-
β-CDLA/ENR	1/1	220	15.6	3.04	fibers	342 ± 59
β-CDLA/ENR	2/1	220	7.8	2.62	fibers	377 ± 92
β-CDLA	-	220	-	1.84	fibers	307 ± 77

**Table 2:** Comparative shift values observed in the ^1^H NMR spectra of ENR and β-CDLA electrospun formulations.

	3a (1H, s)	H2 (1H, s)	H5 (1H, d)	H8 (1H, s)	H1a (1H, m)	H2′, H6′ (4H, m)	H3′, H5′ (4H, m)	H7′ (2H, s)	H1b (2H, d)	H1c (2H, d)	H8′ (3H, t)
ENR	15.20	8.64	7.87–7.84	7.55–7.53	3.83–3.80	3.34–3.32	2.59–2.57	2.44–2.39	1.35–1.30	1.20–1.16	1.07–1.03
1/1 ^1^	15.24	8.67	7.93–7.90	7.58–7.56	3.85–3.83	3.36 *	2.62–2.60	2.47–2.43	1.30 *	1.22–1.17	1.08–1.04
2/1 ^1^	15.24	8.67	7.93–7.90	7.58–7.56	3.85–3.84	3.36 *	2.62–2.61	2.45–2.41	1.30 *	1.24–1.19	1.08–1.04

^1^ Molar ratio of CDLA/ENR; * overlapping with CDLA peaks.

**Table 3:** In vitro antibacterial activity of the β-CDLA/ENR nanofibers.

Sample	Diameter of the Inhibition Zone (mm)		
	*S. aureus*	*E. coli*	*P. aeruginosa*
β-CDLA/ENR 1/1	26.10 ± 0.05	31.70 ± 0.06	17.10 ± 0.05
β-CDLA/ENR 2/1	27.00 ± 0.00	35.10 ± 0.05	18.00 ± 0.00
β-CDLA	0	15.00 ± 0.00	0
ENR	29.00 ± 0.00	34.10 ± 0.05	21.00 ± 0.00
Water/DMSO (3/1 *v*/*v*)	0	0	0
Ciprofloxacin	26.70 ± 0.06	31.00 ± 0.00	32.30 ± 0.57

## Data Availability

The raw/processed data required to reproduce these findings cannot be shared at this time as the data also form part of an ongoing study. However, raw/processed data may be provided upon request.
